# High-risk HPV serotype distribution in cervical cancer: comparative analysis of detection in cervicovaginal secretions and tissue DNA extraction

**DOI:** 10.3332/ecancer.2025.1992

**Published:** 2025-09-18

**Authors:** Abideen A Olayiwola, Yusuf A Oshodi, Oluwarotimi I Akinola, Adekunbiola A Banjo, Kabiru A Rabiu, Ayokunle M Olumodeji

**Affiliations:** 1 Obstetrics and Gynaecology Department, Lagos State University Teaching Hospital, Ikeja, 1-5 Oba Akinjobi Road, PMB 21005, Lagos, Nigeria; 2 College of Medicine, Lagos University Teaching Hospital, Lagos, Lagos State, Nigeria

**Keywords:** human papillomavirus, invasive cervical cancer, high-risk HPV test kit

## Abstract

**Background:**

Cervical cancer is the most common gynaecological cancer. Molecular, clinical and epidemiological studies have shown that high-risk human papillomavirus (HPV) infection plays a causal role in the aetiopathogenesis of cervical carcinoma. This study is to determine the serotype distribution of high-risk HPV deoxyribonucleic acid (DNA) in women with established cervical cancer and compare HPV DNA detected in cervicovaginal secretions with that extracted from formalin-fixed paraffin-embedded tissue (FFPE) section in Lagos State University Teaching Hospital, Southwest, Nigeria.

**Methods:**

This was a comparative study involving subjects with clinical suspicion of cervical cancer. They had examination under anaesthesia, staging and biopsy done, and 44 of the subjects with histologically confirmed cervical cancer were recruited. Their cervico-vaginal secretion samples were taken for high-risk HPV and compared same with high-risk HPV obtained from tissue extraction. The result obtained was analysed using SPSS version 27.

**Result:**

Of 44 subjects with tissue diagnosis of cervical cancer, high risk (hr) – HPV DNA was detected in 38 (86.4%) in both cervicovaginal secretions and the FFPE tissues of the subjects. Of the hr-HPV detected from the FFPE samples, HPV 16 was seen in 27 (61.4%) and HPV 18 in 16 (36.3%) subjects. Other HPV types identified include 45 in 7 (15.9%), HPV 58 in 5 (11.4%), HPV 51 in 3(6.8%), HPV 39 in 2 (4.5%), HPV 66 in 1 (2.3%) and HPV 73 in 1 (2.3%) of cases. There were 16 (36.4%) of single and 22 (50.0%) multiple serotypes of hr-HPV detected. Of the multiple serotypes, two serotypes in 21 (47.3%) cases and three serotypes (16, 18 and 58) in 1 (2.3%) case.

The test kit detected the same number of hr-HPV as in tissue extraction (86.4%); however, with different serotypes. With tissue extraction more (serotypes) HPV 16 and 18 were detected compared to the use of cervicovaginal secretion (*p* = 0.001) and (*p* = 0.046).

The commonest histological type of cervical cancer found among the subjects was squamous cell carcinoma (SCC) 38 (86.4%) and adenocarcinoma 4 (9.2%). Thirty-three subjects (86.8%) of those with SCC were positive for hr-HPV with serotypes 16 and 18, predominating.

**Conclusion:**

The hr-HPV DNA detection rate on cervicovaginal secretion using the test kit is similar to that of tissue extraction FFPE, with serotypes 16 and 18 being predominant. This finding further confirms the reliability of the use of the HPV test kit on cervicovaginal secretion as a screening tool for premalignant and malignant lesions of the cervix.

## Background

Cervical cancer is one of the most preventable malignant diseases. It is the fourth most common cancer in women worldwide, with an estimated 662,044 new cases and 348,709 deaths in 2022 [1]. Geographical disparities in the cervical cancer disease burden are prominent and reflect the availability, coverage and quality of preventive strategies and the prevalence of risk factors [1]. Eight in ten women who die from cervical cancer live in low-income and middle-income countries [2]. Inequalities are widely observed such that incidence rates are declining most rapidly in high-income countries, with some countries moving towards cervical cancer elimination in the coming decades [3]. In contrast, rates are increasing in some Sub-Saharan African settings [2, 4] and or remained relatively stable at high levels in several eastern European and Western Asian countries [2, 4, 5].

Cervical cancer has been reported to be a major public health concern among women of various age groups in Nigeria [6]. In Nigeria, 53.3 million women are estimated to be at risk of developing cervical cancer (International Agency for Research on Cancer/Catalan Institute of Oncology, 2017) with a national standardised prevalence rate of 33.0 per 100,000. Cervical cancer begins when healthy cells in the cervix develop a mutation in their deoxyribonucleic acid (DNA). This results in uncontrollable cellular proliferation, accumulations of abnormal cells, eventually there is a loss of cellular boundary control and then metastasis.

There are multiple risk factors contributing to the development of cervical cancer, which include multiple sexual partners, early sexual activity, sexually transmitted infections such as chlamydia, gonorrhoea, syphilis and human immunodeficiency virus/acquired immunodeficiency syndrome. Weak immune system, smoking, exposure to drugs such as diethylstilboestrol while pregnant are also risk factors.

When premalignant lesions of the cervix and early stage cervical cancer are diagnosed, they can be successfully and effectively treated. Cancers diagnosed in late stages can also be controlled with appropriate treatment and palliative care.

Human papillomavirus (HPV) is mostly sexually transmitted and high-risk HPV DNA is found to be present in 90%–100% of cervical cancer specimens [7]. Within 12–24 months of exposure to the virus, 90% of HPV infections are cleared or become inactive in young women. However, when infections by the high-risk HPV types persist, it then increases the risk of premalignant cervical lesions and progression to cervical cancer [8]. HPV DNA replicates from free DNA in the basal cells of the cervix during the initial period of HPV infection, and then integrates into the host genome as the infection progresses, with subsequent up-regulation of E6 and E7 oncogene expression [9].

There has been an increasing emphasis on HPV type testing in cervical cancer screening and diagnosis. HPV screening is recommended for the further evaluation of abnormal Pap tests or during follow-up after treating precancerous lesions. Several randomised controlled studies have shown that screening for cervical cancer using HPV detection can be more effective than cytology alone [10]. HPV DNA testing is highly sensitive and moderately specific for cervical intraepithelial neoplasia grade 3 or worse, with consistent results across study sites and age groups, including women younger than 35 years [11].

High-risk HPVs can cause several types of cancer (vulvar, vaginal, penile, anal and oropharyngeal). There are about 14 high-risk HPV types, including HPV 16, 18, 31, 33, 35, 39, 45, 51, 52, 56, 58, 59, 66 and 68. Aetiologically, five HPV types (16, 18, 31, 33 and 45) have been linked to 80% of cervical cancers, while the other types contribute the remaining 20% [12]. Two of these, HPV16 and HPV18, are responsible for about 70%–75% of HPV-related cancers [13]. The prevailing serotypes in sub-Sahara Africa, precisely in Nigeria, are 16, 18, 45, 52 and 51 by Kabir *et al* [14].

The detection of high-risk (hr)-HPV serotypes in diagnosed cervical cancer using tissue biopsy is well established as the gold standard in determining the cause in studies done by Abba Kabir *et al* [14], Zohoncon *et al* [16] and Haghshenas *et al* [21], respectively. This, though not routinely done, has been said to be laborious, time-consuming and expensive. In Nigeria, there are limited studies on the testing for hr-HPV serotype distribution in histologically diagnosed cervical cancer patients. Also, there has been no study in Sub-Saharan Africa comparing the detection rate of hr-HPV in cervicovaginal and formalin-fixed paraffin-embedded tissue (FFPE) tissue samples. This study was designed to detect hr-HPV serotypes distribution in the cervicovaginal secretions of confirmed cervical cancer cases, comparing it to those extracted from FFPE tissue samples.

## Materials and methods

The research design was a cross-sectional comparative study between two diagnostic methods in the same participant. The use of a test kit for hr-HPV on cervico-vaginal secretion against the old gold standard method FFFPE–tissue extraction.

The study population was consenting women in the gynaecologic clinic, emergency room, and gynaecological ward who had clinical features suggestive of cancer of the cervix. They had examination under anaesthesia (EUA), staging and biopsy done, and upon review with the histology report, cervico-vaginal secretion was taken for hr-HPV DNA testing in those with a histological diagnosis of cervical cancer.

Consecutive patients with clinical features suggestive of cancer of the cervix who had EUA, staging and biopsy done with eventual histologically confirmed cervical cancer and have satisfied the inclusion criteria (women with clinical features suggestive of cancer of the cervix who had EUA, staging and biopsy done with eventual histologically confirmed cervical cancer) and consented were recruited into the study.

Sample size was determined using the statistical formula for cross-sectional comparative studies [18].

With an estimated non-response rate of 10%, the attrition factor = 10% of 40 = 4.

The sample size was 44.

Convenience sampling method (a non-probability sampling technique) was used to recruit subjects consecutively, using a structured proforma (appendix)containing information on sociodemographic characteristics of the participants, their awareness of cancer of the cervix, sexual and gynaecologic history, risk factors and clinical presentation until the desired sample size was attained.

The patient who qualified and consented had a proforma (data collection tool) to retrieve information on socio-demography, risk factors for cervical cancer, sexual and reproductive history, contraceptive use, among others.

The approval to carry out the study was obtained from the Health Research Ethics Committee of Lagos State University Teaching Hospital, Ikeja and permission was obtained from the department for the use of gynaecological patients.

The participants were informed about the study, as well as their rights and benefits. Written informed consent was obtained through a voluntarily signed consent form. All the participants were able to communicate in English. No participant was coerced in any way to participate in this study, which was at no cost to them. Participants were assured of the confidentiality of their information and all personal identifiers were removed.

Confidentiality was achieved by assigning codes and unique identification numbers to participants. Paper records were stored securely and electronic data were protected with a password.

### Clinical management

Following recruitment of the participants with features suggestive of cervical cancer, the consenting participants were counselled for examination under anaesthesia, staging and biopsy, which were performed in the theatre. Having scrubbed and gowned, the patient was placed in lithotomy position under anaesthesia. After cleaning and draping the patient, the bladder was drained using a metal catheter. A digital vaginal examination was done with a gloved hand to know the extent of the disease, the cervix was reached for and a bimanual examination was done to examine the adnexa and the parametrium. The appropriate speculum size was determined. A recto-vaginal examination was done to check for a cancer-free space, digital rectal examination was done to check the rectal wall and the pelvic side wall. Thereafter, I changed my glove and a speculum vaginal examination was done and a biopsy was taken at the junction between the lesion and healthy tissue using biopsy forceps (Tischler). Haemostasis was secured by pressure packing, and formalin-soaked gauze was used in some cases. The patient was cleaned up and was moved to the recovery room. The biopsy sample was preserved in formalin solution and was sent to the laboratory at the College of Medicine, University of Lagos, for histological analysis. Those who were histologically diagnosed with cervical cancer who presented within 2 weeks for review with their results had a cervical secretion sample collected using a high-risk HPV test kit after obtaining consent. The specimen was obtained by inserting the cyto-brush into the cervical canal and rotating it four times in a clockwise direction to collect all the cervical epithelial cells that adhered to the flat sides of the bristles. The cyto-brush was then inserted into the vial containing preservative fluid. The hybribio cervical collection kit was used. These samples were sent to the laboratory for analysis. Samples from patients, including tissue blocks obtained from biopsies were sent to the molecular laboratory for processing.

The 37 HPV GenoArray Diagnostic Kit (HBGA-37) was used, which is a qualitative polymerase chain reaction (PCR) based *in-vitro* test for the detection and determination of 37 specific HPV DNA in cervical specimens. The assay is optimised to detect 15 high-risk HPV DNA, 6 low-risk HPV DNA and 16 probably low-risk types as shown: 15 high-risk HPV types: HPV 16, 18, 31, 33, 35, 39, 45, 51, 52, 53, 56, 58, 59, 66, 68. Low-risk; 6, 11, 42, 43, 44, CP8304 (81). The probably low-risk are HPV 26, 34, 40, 54, 55, 57, 61, 67, 69, 71, 72, 73, 82, 83, 84.

Confirmed cases of cervical cancer were transferred to the oncology unit of the department for appropriate management based on our unit protocol.

This study involved extracting high-risk HPV DNA from cervicovaginal secretions and tissue samples using specialised kits and molecular techniques. For cervicovaginal secretions, DNA extraction followed a standardised workflow involving cell lysis, PCR amplification and hybridisation with genotype-specific probes using the HybriMax system. The amplified DNA was visualised via colorimetric assays, ensuring precise detection of HPV serotypes. Similarly, genomic DNA from FFPE tissues was extracted using the GMpure FFPE gDNA Kit, following rigorous contamination-prevention protocols. Both methods included stringent quality control measures to ensure reliability.

The processes involved multiple steps, such as controlled sample storage, reagent preparation, DNA amplification and hybridisation. Specific protocols ensured optimal stability of reagents and precision during PCR amplification and hybridisation, supported by temperature-controlled incubations and specialised equipment. Results were interpreted by visualising colorimetric patterns, confirming the presence of high-risk HPV DNA and its serotype distribution. This methodology highlights the robustness of HPV DNA detection from different sample types for reliable cervical cancer diagnosis [19].

### Interpretation of results

The results were interpreted by colour visualisation observed on membranes.

### Dissemination of results

The participants were notified of the diagnosis and referred to the gynaecological-oncology unit of our department, where they were managed according to the unit protocol.

### Data processing and statistical analysis

Data analysis was performed using IBM SPSS version 27. Categorical variables, such as socio-demographic and clinical characteristics, were summarised with frequencies and percentages, while numeric data, like age, were reported using means and standard deviations after confirming normal distribution with the Shapiro–Wilk test. Associations between categorical variables were evaluated using Fisher’s exact test and differences in serotype detection between tissue DNA extraction and hr-HPV test kits were assessed with McNemar’s test. The diagnostic accuracy of the hr-HPV test kit, compared to tissue DNA extraction as the standard, was evaluated using sensitivity, specificity, positive predictive value, negative predictive value and overall accuracy metrics.

### Results

**Figure 1.**
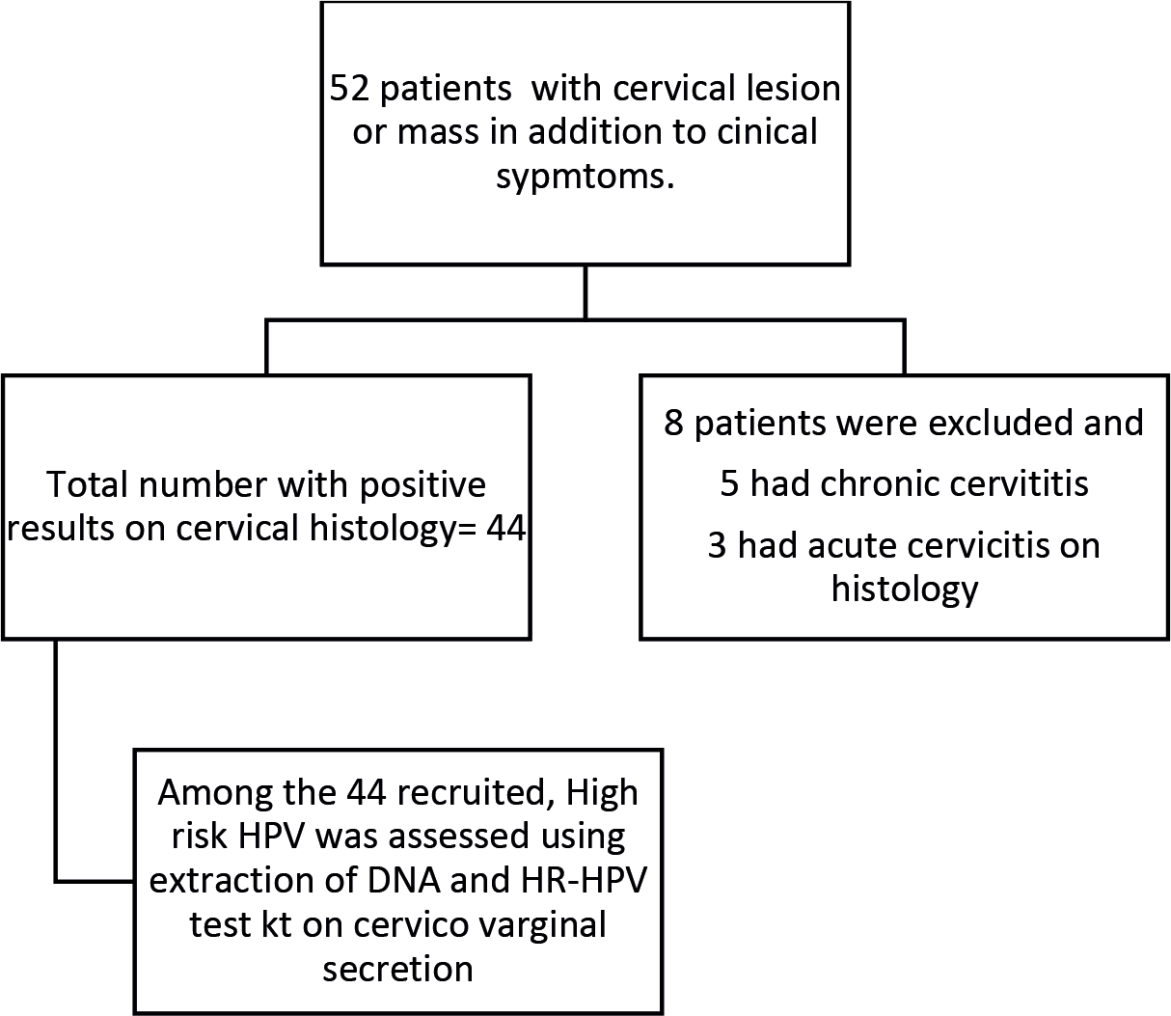
Flow chart showing recruited participants.

The study flow chart is shown in Figure 1. The mean age of the patients was 53.46 ± 10.9 years (minimum of 36 years and maximum of 95 years).

### Sociodemographic characteristics

Majorly, the subjects were above 40years of age, 38 (86.4%) and only 6 (13.6%) were 40 years and below. Fourteen of the subjects (31.8%) were between the ages of 41 and 60 years, while 10 (22.7%) subjects were above 60 years.

Twelve (27.3%) subjects had 4 and more parous experience while 30 (68.2%) had between 1 and 3 parous experience. Only 2 (4.5%) of the subjects were nulliparous. Twenty-six of them (59.1%) had secondary and tertiary levels of education, 14 (31.8%) had primary level of education, while 4 (9.1%) had no formal education. Most of the subjects, 27 (61.4%) were Christians, while 16 (36.4%) and 1 (2.7%) were of Islam and Traditional religion, respectively. The majority of the subjects were Yoruba 30 (68.2%), while 9 (20.5%) were Igbo, 1 (2.3%) was Hausa and 4 (9.1%) belonged to other tribes Table 1.

Most of the subjects, 43 (93.2%) were not aware of cervical cancer, only 1 (2.3%) was aware, who happened to be the only subject that had been screened with VIA (visual inspection with acetic acid). None of the subjects had received the HPV vaccine, while 6 (13.6%) of them had a family history of cervical cancer, as demonstrated in Table 1.

Twenty-six subjects (53.1%) attained coitarche at less than 20 years, it was at between 20 and 29 years in 16 (36.4%) of them, and at 30 and above in 2 (4.5%) of them, respectively. Most of the subjects, 31 (70.5%) had between 2 and 5 lifetime sexual partners, 5 (11.4%) had more than 5 lifetime sexual partners, while 8 (18.2%) had only one lifetime sexual partner. Twenty-five (58.6%) of them had previously used contraceptives and the commonly used contraceptive was condom 18 (72.0%), followed by injectable 4 (16.0%), intrauterine contraceptive device 3 (12.0%) and oral contraceptive pills 2 (8.0%) Table 2.

Most of the subjects, 33 (75.0%) complained of postcoital bleeding, 21 (47.7%) had postmenopausal bleeding and 17 (38.6%) had intermenstrual bleeding, while 32 (72.7%) of them presented with foul-smelling vaginal discharge. There was weight loss in 19 (43.2%) and pedal oedema in 2 (4.5%) of the subjects Table 3.

In terms of the stage of the disease at presentation, most of the subjects, 30 (68.2%) presented at stage 2b. Nine (20.5%) presented at stage 3a, 2 (4.5%) presented at 3b, while 3 (6.8%) presented at stage 4a Table 1.

On the detection of hr-HPV in histologically diagnosed cervical cancer cases.

The commonest histological type of cervical cancer found among the subjects was squamous cell carcinoma (SCC) 38 (86.4%), while there were adenocarcinoma 4 (9.2%), adenosquamous carcinoma 1 (2.3%) and small cell neuroendocrine carcinoma 1 (2.3%), respectively. Of the 38 SCC, there was detection of hr-HPV in 33 (86.6%) cases, with most serotypes being 16 and 18, while 5 (13.2%) cases were negative for hr-HPV. Of the 4 adenocarcinoma cases, 3 (75.0%) were positive for hr-HPV, while 1 (25.0%) case was negative for hr-HPV. Only one case each of adenosquqamous and small cell neuroendocrine carcinoma was recorded and were both positive for hr-HPV Table 4.

**Figure 2.**
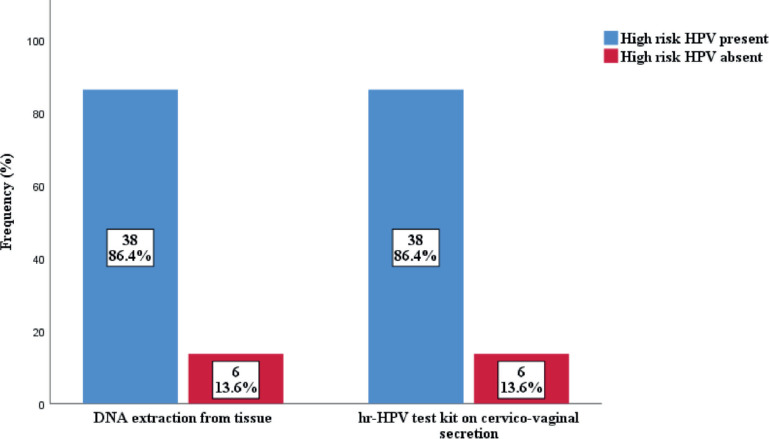
Detection of high risk HPV among subjects.

**Figure 3.**
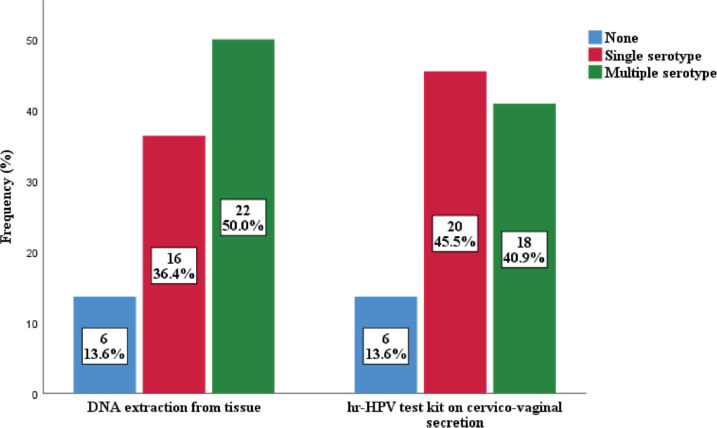
Combinfation of serotypes among subjects.

The prevalence of high-risk HPV detected in cervicovaginal secretions of the subjects was found to be similar to that extracted from the FFPE tissue samples (Figure 2). Thirty-eight cases (86.4%) were positive for hr-HPV, while 6 (13.6%) were negative in both samples. In the FFPE sample, the predominant serotypes detected were HPV 16 and 18, accounting for 61.4% and 36.3%, respectively. Others were HPV 45 (15.9%), HPV 58 (11.4%), HPV 51 (6.8%), HPV 39 (4.5%) and HPV 66 (2.3%). There were single and multiple serotypes of hr-HPV detected in some cases, 16 (42.1%) and 22 (57.9%) of single and multiple serotype, respectively. Of the multiple serotypes detected, 21 (95.5%) were two, while 1 case (4.5% had 3 serotypes. Whereas in the cervicovaginal secretions, the detection of hr-HPV 16 and 18 was 40.9% and 27.3%, respectively. Other detected hr-HPV were HPV 45 (15.9%), HPV 58 (11.4%), HPV 51 (9.4%), HPV 35 (6.8%), HPV 39 (4.5%) and HPV 66 (2.3%) Table 5. Single and multiple serotypes detection was also recorded. There were 20 (45.5%) single infections and 18 (40.9%) multiple infections. Of the multiple serotype detected, 17 (38.6%) were double and 1 (2.3%) case was triple Figure 3.

In establishing the comparison between the detection rate of the test kit on cervicovaginal secretion versus DNA extraction from tissue. Of the detected 8 hr-HPV using both methods, there was a significant association in the detection of HPV 16 (*p* < 0.001) and HPV 18 (*p* = 0.046) using the Mcnemar test. There were no significant associations in the detection of HPV 35 (*p* = 0.087), HPV 39 (*p* = 1.000), HPV 45 (*p* = 1.000), HPV 51 (*p* = 0.877), HPV 58 (*p* = 1.000) and HPV 66 (*p* = 1.000) using both methods Table 5.

To determine the level of agreement between hr-HPV test kit on cervico-vaginal secretion in detecting hr-HPV serotypes and that of DNA extraction from cervical cancer tissue. There was total agreement in 30 (68.2%), partial agreement in 8 (18.2%) and no agreement in 6 (13.6%) of cases using both methods.

In determining the association between high-risk HPV status and histological type of cervical cancer. There was no significant association between the hr-HPV status and histological types of cervical cancer (x^2^- 0.762) (*p* = 0.859) Table 4.

On the association between high-risk HPV status and sociodemographic characteristics/lifestyle characteristics, there was no association between the age, parity, educational status of the subjects and the hr-HPV status using Fisher's exact (*p* = 0.982) (*p* = 0.409) (*p* = 0.776), respectively. Concerning their previous awareness on cervical cancer (*p* = 0.303), previous screening (*p* = 0.565), previous vaccine (*p* = 0.688), family history (*p* = 0.816), age at coitarche (*p* = 0.307), number of sexual life partners (*p* = 0.618), types of contraceptives used (*p* = 0.600) and subject’s hr-HPV status, there was no association Table 3.

On the association between the hr-HPV status and clinical characteristics of the subjects, there was no significant association between postcoital bleeding (*p* = 0.612), postmenopausal bleeding (*p* = 0.060), intermenstrual bleeding (*p* = 0.538), foul-smelling vaginal discharge (*p* = 0.720) and the hr-HPV status of the subjects Table 3.

### Discussion

The study was conducted to compare the detection rate of high-risk HPV using a test kit against the standard tissue extraction. High-risk HPV was detected in 38 (86.4%) of the 44 cervical cancer cases using DNA extraction from tissue samples. This is comparable to what was found in Lagos (85.6%) [20] and but not comparable to findings in Maiduguri (69.8%) [14]. This may be due to the different population studied or the mode of tissue preservation. Our subjects were predominantly above 40years of age 38 (86.4%), as only 6 (13.6%) were 40 years and below, the detection of hr-HPV was higher above 40 years of age. This is in conformity with the findings in Lagos [20] and Iran [21], where most of their hr-HPV detected were from subjects above 40years of age. Of the 38 SCC, there was detection of hr-HPV in 33 (86.6%)

cases, with most serotypes being 16 and 18. This is higher than the findings in a study in Lagos [20] where hr-HPV 16 and 18 were recorded in 69.6% of their cases of SCC. Of the 4 adenocarcinoma cases, 3 (75.0%) were positive for hr-HPV, while 1 (25.0%) case was negative for hr-HPV. This is in contrast to the Lagos study, where multiple detection of hr-HPV was recorded in their 6 cases of adenocarcinoma. Only one case each of adenosquqamous and small cell neuroendocrine carcinoma was recorded and were both positive for hr-HPV. This is similar to findings in Lagos [20], where they detected hr-HPV in all their cases of adenosquamous carcinoma and small cell neuroendocrine carcinoma. This may be due to the high association of hr-HPV with cervical cancer.

Several variables (age, parity, educational status, age at sexual debut, lifetime sexual partners and family history of cervical cancer) observed in this study revealed no significant association with HPV status. This is similar to the findings in the Lagos study [15]. This may be due to the population studied and the sample size.

The detection rate of HPV 16 and 18 with tissue extraction was 61.4% and 36.3%, respectively. Making 16 and 18 the most common HPVs detected in this study. This is in agreement with the global trend and conforms to some studies [13, 14, 22, 20]. In contrast, serotypes 16 (63.4%) and 66 (4.6%) were the predominant strains found in Brazil [23]. Other HPV found in this study were HPV 45 (15.9%), HPV 58 (11.4%), HPV 51 (6.8%), HPV 39 (4.5%), HPV 61 (2.3%) and HPV 66 (2.3%), indicating the diverse nature of high-risk HPV in our environment. Variations were observed in our findings compared to those of Kabir *et.al* [17], where HPV 31, 35 and 73 were part of the serotypes found in tissue extraction of DNA FFPE [13]. There were single 16 (36.4%) and multiple 22 (50.0%) serotypes of hr-HPV detected in some cases. This is similar to the findings of a similar study in Maiduguri, where single 17 (38.6%) and multiple 27 (61.4%) serotypes were recorded [14]. Of the multiple serotypes detected in this study, two serotypes were found in 21 (47.7%) cases, while three serotypes were found in 1 (2.3%) case. In contrast to a study where 9 (20.5%) of double and 10 (22.7%) of triple serotypes were recorded [14]. The difference may be due to the study population and geographical location.

In the use of high-risk HPV test kits on cervico-vaginal secretion, 38 (86.4%) of the 44 cervical cancer cases were hr-HPV positive. Five (HPV types 16, 18, 35, 45 and 58) out of the eight (HPV types 16, 18, 31, 33, 35, 45, 52 and 58) globally most common cervical HPV types among women were detected using this method. The distribution of hr-HPV detected was HPV 16 (40.9%), HPV 18 (27.3%), HPV 45 (15.9%), HPV 58 (11.4%), HPV 51 (9.1%), HPV 35 (6.8%), HPV 39 (4.3%) and HPV 66 (2.3%).

More cases of HPV 16 and 18 were significantly detected in FFPE tissue samples compared to cervicovaginal secretions (*p* < 0.001) (*p* = 0.046). Out of the eight (HPV types 16, 18, 31, 33, 35, 45, 52 and 58) global most common HPV types among women, [24, 25] 4 serotypes (HPV 16, 18, 45 and 58) were detected in FFPE samples while 5 serotypes (HPV 16, 18, 35, 45 and 58) were detected in cervicovaginal secretions. The degree of detecting multiple HPV serotypes from the various samples varied. Detection of multiple HPV types was higher in FFPE tissue samples (50.0%) compared with the rate in cervico-vaginal secretion (40.9%). This is higher than what was found in Lagos [20], where the rate for multiple detection in tissue extraction was 4.4%, but similar to the findings of two other studies [26, 27]. Their ability for single detection of HR-HPV in this study was 36.4% and 45.5%, respectively. There was total agreement of both methods in 62.8% of cases, both methods were able to detect HPV types 16, 18, 39, 45, 51, 58 and 66 simultaneously. While partial agreement and no agreement accounted for 18.2% and 13.6%, respectively.

The use of the HPV kit on cervico-vaginal secretion to detect hr-HPV histologically diagnosed cervical cancer demonstrated very high sensitivity, 94.7% but low specificity, 66.7% in this study.

### The limitation of the study

Limited literature was found on previous studies, small sample size, which may not make the result generalisable and the examination under anaesthesia was unconventional as there was no cystoscopy or proctosigmoidoscopy done.

### Strength of the study

i) Study assessing the presence of hr-HPV DNA in cervical cancer are scarce, more so in our environment. This research provides current evidence of hr-HPV serotypes as described above.
ii) The innovation of a new method of isolating hr-HPV-DNA in lieu of the standard tissue extraction in established cancer of the cervix with a comparable detection rate, though with different strains. While tissue extraction detected more of HPV16 and 18, the test kit detected more hr-HPV of different serotypes.
iii) Use of relatively fresh cervical biopsy tissues for DNA extraction against previous studies, which used tissues from archives after a long storage period.
iv) This new approach to detection using a test kit with easier processing, short turn-around time to getting result, facilitating earlier intervention, may be adopted as a first line after validation using a larger sample size.

## Conclusion

The study identified the use of the HPV test kit on cervicovaginal secretion as a useful tool in detecting similar cases as in tissue extraction, though with different serotypes. HPV 16 and 18 were the predominant serotypes, with tissue extraction detecting more serotypes than the test kit on cervicovaginal secretion. This finding further confirms the reliability of the use of the HPV test kit on cervicovaginal secretion as a screening tool for premalignant and malignant lesions of the cervix.

## List of abbreviations

DNA, Deoxyribonucleic acid; EUA, Examination under anaesthesia; FFPE, Formalin fixed paraffin embedded tissue; HIV, Human immunodeficiency virus; HPV, Human Papilloma Virus; hr, High risk; ICO, Catalan Institute of Oncology; PCR, Polymerase chain reaction; SCC, Squamous cell carcinoma.

## Conflicts of interest

There was no conflict of interest.

## Funding

No external fund was received for the work.

## Figures and Tables

**Table 1.** Characteristics of study participants.

Variables	Frequency (*n* = 44)	Percentage (%)
Age group (Years) <40 41–50 51–60 >60 Mean ± SD*	6 14 14 10 53.46 ± 10.9	13.6 31.8 31.8 22.7
Parity 0 1–3 ≥4	2 30 12	4.5 68.2 27.3
Educational status None Primary Secondary Tertiary	4 14 16 10	9.1 31.8 36.4 22.7
Coitarche <20years 20–29 years 30 and above	26 16 2	59.1 36.4 4.5
Number of total life sexual partners 1 2–5 >5	8 31 5	18.2 70.5 11.4
Used contraception before Yes No	25 19	56.8 43.2
Types of contraception used (n = 25) Condom Injectable IUD OCP	18 4 3 2	72.0 16.0 12.0 8.0
Stage of disease 2b 3a 3b 4a	30 9 2 3	68.2 20.5 4.5 6.8

SD* = Standard deviation

**Table 2.** Association between high-risk HPV status and socio-demographic characteristics/lifestyle characteristics.

	HPV		Total	Fisher exact	p-value
	Positive (*n* = 38) *n* (%)	Negative (*n* = 6) *n* (%)			
Age group (Years) <40 41–50 51–60 >60	5 (83.3) 12 (85.7) 12 (85.7) 9 (90.0)	1 (16.7) 2 (14.3) 2 (14.3) 1 (10.0)	6 (100.0) 14 (100.0) 14 (100.0) 10 (100.0)	0.169	0.982
Parity 0 1–3 ≥4	2 (100.0) 26 (86.7) 10 (83.3)	0 (0.0) 4 (13.3) 2 (16.7)	2 (100.0) 30 (100.0) 12 (16.7)	0.412	0.814
Educational status None Primary Secondary Tertiary	4 (100.0) 12 (85.7) 13 (81.3) 9 (90.0)	0 (0.0) 2 (14.3) 3 (18.8) 1 (10.0)	4 (100.0) 14 (100.0) 16 (100.0) 10 (100.0)	1.104	0.776
Heard of cervical cancer before Yes No	2 (66.7) 36 (87.8)	1 (33.3) 5 (12.2)	3 (100.0) 41 (100.0)	1.061	0.303
Ever been screened for cervical cancer Yes No	2 (100.0) 36 (85.7)	0 (0.0) 6 (14.3)	2 (100.0) 42 (100.0)	0.331	0.565
Ever had HPV vaccine Yes No	1 (100.0) 37 (86.0)	0 (0.0) 6 (14.0)	1 (100.0) 43 (100.0)	0.162	0.688
Family history of cervical cancer Yes No	5 (83.3) 33 (86.8)	1 (16.7) 5 (13.2)	6 (100.0) 38 (100.0)	0.054	0.816
Coitarche <20years 20–29 years 30 and above	23 (88.5) 14 (87.5) 1 (50.0)	3 (11.5) 2 (12.5) 1 (50.0)	26 (100.0) 16 (100.0) 2 (100.0)	2.360	0.307
Number of total life sexual partners 1 2–5 >5	7 (87.5) 26 (83.9) 5 (100.0)	1 (12.5) 5 (16.1) 0 (0.0)	8 (100.0) 31 (100.0) 5 (100.0)	0.962	0.618
Used contraception before Yes No	21 (84.0) 17 (89.5)	4 (16.0) 2 (10.5)	25 (100.0) 19 (100.0)	0.275	0.600

**Table 3.** Association between high-risk HPV status and clinical characteristics.

	HPV		Total	Fisher exact	*p*-value
	Positive (*n* = 38) *n*(%)	Negative (*n* = 6) *n*(%)			
Post-coital bleeding Yes No	29 (87.8) 9 (81.8)	4 (12.1) 2 (18.2)	33 (100.0) 11 (100.0)	0.257	0.612
Post-menopausal bleeding Yes No	16 (76.2) 22 (95.7)	5 (23.8) 1 (4.3)	21 (100.0) 23 (100.0)	3.530	0.060
Inter-menstrual bleeding Yes No	14 (82.4) 24 (88.9)	3 (17.6) 3 (11.1)	17 (100.0) 27 (100.0)	0.378	0.538
Foul smelling vaginal discharge Yes No	28 (87.5) 10 (83.3)	4 (12.5) 2 (16.7)	32 (100.0) 12 (100.0)	0.129	0.720
Weight loss Yes No	16 (84.2) 22 (88.0)	3 (15.8) 3 (12.0)	19 (100.0) 25 (100.0)	0.132	0.717
Abdominal mass Yes No	8 (88.9) 30 (85.7)	1 (11.1) 5 (14.3)	9 (100.0) 35 (100.0)	0.061	0.805
Pedal oedema Yes No	2 (100.0) 36 (85.7)	0 (0.0) 6 (14.3)	2 (100.0) 42 (100.0)	0.331	0.565

**Table 4.** Association between HPV infection and cervical cancer histological types.

	Present of HPV DNA (*n* = 38)	Absent of HPV DNA (*n* = 6)	Total	*χ* ^2^	*p*-value
Squamous cell carcinoma	33 (86.8)	5 (13.2)	38 (100.0)	0.762	0.859
Adenocarcinoma	3 (75.0)	1 (25.0)	4 (100.0)		
Adenosquamous carcinoma	1 (100.0)	0 (0.0)	1 (100.0)		
Small cell neuroendocrine carcinoma	1 (100.0)	0 (0.0)	1 (100.0)		

**Table 5.** Comparison ofHPV Serotypes results using both diagnosed methods (DNA extraction from cervical tissue and hr-HPV test kit on cervico-vaginal secretion) study population.

	Diagnostic method		Mcnemar test	*p*-value
	DNA extraction from cervical tissue	hr-HPV test kit		
HPV serotypes	No (%)	No (%)		
HPV 16	27 (61.4)	18 (40.9)	17.392	<0.001*
HPV 18	16 (36.3)	12 (27.3)	2.000	0.046*
HPV 35	0 (0.0)	3 (6.8)	1.582	0.087
HPV 39	2 (4.5)	2 (4.5)	0.000	1.000
HPV 45	7 (15.9)	7 (15.9)	0.000	1.000
HPV 51	3 (6.8)	4 (9.1)	0.492	0.877
HPV 58	5 (11.4)	5 (11.4)	0.000	1.000
HPV 66	1 (2.3)	1 (2.3)	0.000	1.000
Negative	6 (13.6)	6 (13.6)	0.000	1.000

* Statistically significant
